# Flow Diversion for Endovascular Treatment of Intracranial Aneurysms: Past, Present, and Future Directions

**DOI:** 10.3390/jcm13144167

**Published:** 2024-07-16

**Authors:** Michael Gaub, Greg Murtha, Molly Lafuente, Matthew Webb, Anqi Luo, Lee A. Birnbaum, Justin R. Mascitelli, Fadi Al Saiegh

**Affiliations:** Department of Neurosurgery, University of Texas Health Science Center at San Antonio, 7703 Floyd Curl Drive, MC 7843, San Antonio, TX 78229, USA; gaub@uthscsa.edu (M.G.); murthag@uthscsa.edu (G.M.); lafuentem@uthscsa.edu (M.L.); webbmr@uthscsa.edu (M.W.); luoa@uthscsa.edu (A.L.); birnbaum@uthscsa.edu (L.A.B.); mascitelli@uthscsa.edu (J.R.M.)

**Keywords:** flow diversion, intracranial aneurysms, wide-neck aneurysms, PUFS trial, WEB-IT trial

## Abstract

Flow diversion for intracranial aneurysms emerged as an efficacious and durable treatment option over the last two decades. In a paradigm shift from intrasaccular aneurysm embolization to parent vessel remodeling as the mechanism of action, the proliferation of flow-diverting devices has enabled the treatment of many aneurysms previously considered untreatable. In this review, we review the history and development of flow diverters, highlight the pivotal clinical trials leading to their regulatory approval, review current devices including endoluminal and intrasaccular flow diverters, and discuss current and expanding indications for their use. Areas of clinical equipoise, including ruptured aneurysms and wide-neck bifurcation aneurysms, are summarized with a focus on flow diverters for these pathologies. Finally, we discuss future directions in flow diversion technology including bioresorbable flow diverters, transcriptomics and radiogenomics, and machine learning and artificial intelligence.

## 1. Introduction

The rise of flow diversion over the last two decades fueled a revolution in the management of patients harboring intracranial aneurysms (IAs) [1]. Whilst traditional IA repair techniques have focused on treatments delivered *directly* to the aneurysm sac itself, such as the use of surgical clipping for aneurysm neck reconstruction or the endovascular deployment of detachable coils within the aneurysm sac, the implementation of “flow diverting” devices represented a paradigm shift where aneurysm exclusion from the circulation is driven *indirectly* by remodeling the parent artery. Flow diverters (FDs) promote aneurysm exclusion from the circulation via two main mechanisms: (1) by attenuating blood flow into the aneurysm sac leading to intrasaccular stasis and thrombosis, and (2) by promoting the formation of a neointimal endothelial layer across the aneurysm neck [2]. As such, IAs are treated without ever manipulating the aneurysm sac, a concept unique to both surgical and endovascular treatment modalities.

The term “flow diverters” was coined in 2004 as the title of an NIH grant from the University of Miami to study minimized porosity endoluminal stents as a potential cure for IAs [3]. Commercial entities soon began in vivo human trials for these new devices, with the Silk flow diverter (Balt; Montmorency, France) first gaining CE approval in Europe in 2008, [4] followed by the Pipeline Embolization Device (PED, Medtronic; Minneapolis, Minnesota) receiving first FDA FD approval in the United States in 2011 [5,6]. The pivotal studies [7,8,9] leading to FD regulatory approval have focused on the treatment of wide-neck aneurysms (WNAs). These “difficult to treat” aneurysms are characterized by their wide aneurysm neck, with criteria for WNAs including aneurysms with one of three features: (1) a neck diameter ≥ 4 mm, (2) a dome-to-neck ratio < 2, or (3) both [10]. WNAs pose treatment challenges via other reconstructive endovascular methods due to the tendency of coils to prolapse or migrate from the aneurysm sac. This is especially the case when WNAs are very large (>2 cm in maximum diameter) or giant (>2.5 cm), where deconstructive techniques such as parent vessel sacrifice are often needed [11]. As such, FD first gained popularity in WNA treatment by eliminating the need for intrasaccular coils or parent artery occlusion. 

In this review, we discuss the history and development of FDs, highlighting the PED and its subsequent generations. A basic description of the mechanisms underlying FDs’ biophysical action and research tools driving translational research is provided. We summarize current on-label indications for FD use in treating IAs. We also delve further into therapeutic dilemmas associated with FD use in off-label pathologies, such as for distal and ruptured aneurysms. The Woven EndoBridge Device (WEB, MicroVention; Aliso Viejo, CA, USA) is introduced as the prototypical intrasaccular flow diverter (ISFD), a more recent addition to the flow diversion portfolio [12]. Finally, future directions are presented, including bioresorbable flow diverters (BRFDs), transcriptomics and radiogenomics, robotic delivery systems, and artificial intelligence and machine learning (ML). Clinical equipoise about the optimal treatment of IAs remains steadfast, with flow diversion a crucial component of the neurointerventional armamentarium.

## 2. History and Development

IAs present with diverse appearances but similar underlying pathophysiology; a weakness or defect in one or multiple vessel wall layers leads to progressive dilation of the affected vascular segment [13]. The most commonly encountered aneurysm morphology is the saccular form, commonly referred to as berry aneurysms, where focal weakness in the tunica media and internal elastic lamina produce a balloon-like outpouching of the vessel side wall, most commonly at bifurcation or daughter vessel branch points. Other non-saccular aneurysm morphologies, such as fusiform and blister, are generally considered more difficult to treat [14,15].

Raymond et al. conducted a retrospective review of 501 saccular aneurysms previously treated with standalone coil embolization and found that large aneurysm diameter (≥10 mm), wide aneurysm neck (>4 mm), and initial incomplete occlusion of either the aneurysm sac or neck were risk factors for major aneurysm recurrence following treatment [16]. Given the high rates of rupture of untreated large and giant aneurysms over time (14.5–50.0% 5-year cumulative risk) [17] and the considerable morbidity and mortality associated with aneurysmal subarachnoid hemorrhage (SAH) [18,19], investigators sought to find more durable endovascular treatment solutions for these difficult-to-treat large and wide-neck aneurysms that are poorly amenable to standalone coiling [20]. The idea that aneurysms arise from diseased parent vessel segments and that treatments aimed at repairing these segments could effectively treat IAs emerged nearly 30 years ago, a concept known as parent vessel remodeling [3]. 

Reports of endoluminal bioprosthetics capable of altering intrasaccular fluid dynamics and reconstructing neck angioarchitecture surfaced in the early 1990s [21]. Funding [22] to develop large and wide-neck aneurysm models capable of testing various FD designs soon followed (e.g., rabbit elastase model [23], human blood loop [24,25]). With the integration of other bioengineering research tools, such as computational fluid dynamics (CFD) [26], optical coherence tomography (OCT) [27], and scanning electronic microscopy (SEM) [28], fundamental flow diverter design principles were elucidated. 

In brief, stent design involves several basic parameters such as architecture (honeycomb vs. braided), porosity and metal coverage (inverse properties, reflective of the total area of stent strand or cell coverage along the vessel wall), pore density (reflective of the degree of spacing between adjacent strands or cells), and material (commonly a metal alloy such as elgiloy or nitinol). In contrast to laser-cut stents used for coiling support (e.g., Neuroform, Stryker Neurovascular; Fremont, CA, USA) which feature a honeycomb structure with low metal coverage (6–12%) and high porosity (88–94%), early FD studies showed that stents with a braided design with minimized porosity and metal coverage around 30–35% produced the maximal intended effect of aneurysm flow diversion and perforator preservation. In short, this combination results in just enough flow diversion to produce intra-aneurysmal stasis and thrombosis without occluding important adjacent vessels supplying normal brain that the stent may cover [29,30]. As device and delivery systems were refined, first-generation FD models became ready for human clinical trials near the end of the early 2000s [5]. 

## 3. Pipeline Embolization Device—The Prototypical Flow Diverter in the USA

The Pipeline Embolization Device was the first flow-diverting device to achieve widespread use in North America. The PED device and associated delivery system have undergone four iterations since FDA premarket approval in 2011. Pipeline Classic (first generation) and Pipeline Flex (second generation) feature the same bare-metal, 48-strand stent design with an upgraded delivery system introduced with Flex in 2014 to facilitate better handling, deployment, and device recapture [31]. PED is a braided stent composed of cobalt–nickel–chromium alloy strands that are not connected or attached in any way. This design allows the metal braids to slide over one another and conform to different vessel diameters and shapes. In comparison to traditional coil support stents (e.g., Stryker Neuroform) that demonstrate a laser-cut open- or closed-cell design with low metal coverage of around 6–12% and minimal flow diversion properties, FDs are braided stents with high metal coverage to maximize the flow diversion effect. Unlike coil support stents that are designed to maximize intrasaccular coil packing, FDs are unable to be crossed by microcatheters, requiring aneurysm access via a microcatheter before FD deployment.

The design and flexibility of the PED generates a range of metal coverage or porosity levels when deployed endovascularly. As the metal braids slide over one another, the PED’s crisscrossing wire angle and length change. As per manufacturing data, this results in a proposed 30–35% metal coverage at nominal diameter; however, several considerations come into play when deploying a device. The actual metal coverage in situ is often less than the manufacturer’s value based on vessel diameter and vessel curvature; additionally, the presence of multiple devices deployed across a segment also variably impacts metal coverage. PED is delivered through a microcatheter using varying degrees of pushing the delivery system while unsheathing the catheter. Many considerations and nuances of the PED’s deployment are beyond this summary’s scope. Regardless, Jabbour et al. found that operator efficiency with FD deployment improves with increased operator experience, demonstrating a “learning curve” to proficient use [32]. 

Pipeline Flex with Shield technology (third generation) is a surface-modified version of PED Classic released in 2015 and features a phosphorylcholine (PC)-coated endoluminal surface to reduce thrombogenicity [33]. The covalently bound PC layer mimics the cell surface of erythrocytes, leading to improved device hemocompatibility. Pipeline Vantage (PEDV, fourth generation) is the latest iteration of the PED device released in 2020. PEDV includes Shield technology but features an upgraded stent design with higher pore density, thinner braid strands, and drawn-filled tubing (DFT) wires to enhance device endothelialization and visualization [34,35].

## 4. Mechanisms of Action and Fate of Jailed Arterial Branches

Fundamentally, all FDs produce aneurysm decoupling and exclusion from the circulation through two mechanisms:

### 4.1. Reduction in Intrasaccular Blood Flow Leading to Intra-Aneurysmal Stasis and Thrombosis 

Blood flow into aneurysms is a complex but predictable process [36]. Flow into the aneurysm sac occurs in a concentrated, high-velocity manner at the distal aneurysm neck. After entering the aneurysm sac, blood swirls around concentrically and then returns to the parent vessel in a diffuse, low-velocity fashion. After FD deployment, a reduction in the mean and peak kinetic energy of blood flow at the aneurysm neck is seen, leading to stasis within the aneurysm sac and, ultimately, thrombosis, as governed by the principles of Virchow’s triad [37,38]. Notably, stasis is a strong impetus for intrasaccular thrombosis despite the concurrent use of dual antiplatelet therapy (DAPT). CFD studies demonstrate the ability of FDs to elicit these aneurysmal flow alterations while maintaining normal parent vessel flow velocities throughout the cardiac cycle. In vivo CFD analysis distinguishes aneurysms destined for either obliteration or residual remnant at follow-up by demonstrating either elimination or persistence of a high-flow intrasaccular vortex following FD placement [23,26,29,30].

While some report benefits via expedited IA occlusion times [39], the addition of intrasaccular coils has not been found to substantially improve overall long-term occlusion rates in large clinical series [40]. However, in the senior author’s experience, loose coiling, in addition to FD of giant or near-giant aneurysms, is advised, as sole FD can cause a rapid thrombosis and inflammatory reaction that could lead to hemorrhagic complications. A defining feature of IAs treated with flow diversion is that, unlike other endovascular modalities, occlusion rates increase with time. For those with available follow-up, an extensive registry of over 1000 aneurysms found progressive complete occlusion rates of 75.8% (588/776), 92.9% (300/323), and 96.4% (159/165) at 1 year, 2–4 years, and >5 years, respectively; long-term angiographic outcomes for those without follow-up are unclear [41]. Evidence of progressive complete occlusion rates following FD therapy have been demonstrated in multiple studies, including the Pipeline for Uncoilable or Failed aneurysms trial (see below; 73.6% at 6 months to 86.8%, 93.4%, and 95.2% at 1, 3, and 5 years, respectively) and the single-institution, 10-year PED experience at the University at Buffalo (complete occlusion rates at 1, 3, 5, and 10 years of 94.7%, 96.6%, 96.0%, and 100%, respectively) [42,43]. However, immediate occlusion rates remain a concern, especially in ruptured aneurysms. As such, a “plug and pipe” approach may be considered where aneurysm dome protection with coils is later followed by FD placement in a staged procedure [44]. 

### 4.2. Provision of a Biocompatible Scaffold Promoting Neointimal Formation across the Aneurysm Neck

Experimental studies demonstrate the histologically proven formation of a new endothelial layer across the aneurysm neck following FD placement [1,45]. FD braid strands provide the necessary scaffold for migrating adjacent parent vessel endothelial progenitor cells, which ultimately form the non-permeable tissue layer, leading to aneurysm decoupling from the circulation. In fact, it is this neo-endothelialization that guarantees permanent exclusion from the blood circulation once an aneurysm is occluded. Device apposition to parent vessel walls appears particularly important in this process as the endothelium is primarily derived from migrating epithelial progenitor and smooth muscle cells from the surrounding parent vessel through local paracrine signaling [46]. This mechanism putatively explains the lower rate of aneurysm occlusion for fusiform and other non-saccular aneurysms where wall apposition may be suboptimal [47,48,49]. Furthermore, FD devices are designed to optimize neointimal formation through their design (strut thickness and braid angle) and function (reduction in wall shear stresses and intrasaccular flow); OCT and post-mortem histologic studies have indeed demonstrated heterogenous neointimal growth with coil support stents where the flow diversion effect is minimal [27,50]. 

Device thrombogenicity appears to play an essential role in this process. Beyond acute ischemic and thromboembolic complications from in-stent macro-thrombosis and occlusion, microthrombus formation leads to uneven, heterogeneous neointimal formation and parent vessel stenosis. Effective antiplatelet (AP) therapy is thus mandatory for all devices to date while neointimal formation transpires, with most patients remaining on DAPT for between 3 and 6 months post-placement [51]. As discussed below, newer luminal surface coating technologies designed to reduce stent thrombogenicity have shown more homogenous neointimal formation through reduced in-stent microthrombus formation [27]. Surface modification of metal devices and the future development of BRFDs may ultimately lead to a lessened duration and magnitude (dual vs. single agent) of AP therapies in the future [52]. 

### 4.3. The Fate of Jailed Arterial Side Branches and Covered Perforators Is a Critical Consideration with FD Placement

Covered arterial branches occlude subsequent stent placements by four main mechanisms: (1) critical metal coverage of the ostium producing immediate obstruction, (2) neointimal formation across the ostium producing delayed obstruction, (3) in-stent thrombosis with variable timing of onset, or (4) longitudinal redistribution of atherosclerotic plaque leading to immediate obstruction via the “snowplowing effect” [53]. While reduced mean and peak inflow systolic velocities have been observed in animal models [54], clinically, studies have shown low rates of side branch occlusion and even lower rates of symptomatic neurologic deficit following jailing of arterial side branches with a flow-diverting device. 

In 1152 supraclinoid ICA aneurysms treated with FDs, Cagnazzo et al. showed low rates of benign and symptomatic occlusion in covered ophthalmic (OphA; 5.9%, 0.8%), posterior communicating (PCoA; 20.7%, 0%), and anterior choroidal (AChA; 1%, 1%) arteries [55]. Iosif et al. found similar findings in 63 FD procedures involving the middle cerebral artery (MCA) bifurcation and M1-segment lenticulostriates, with 14.3% asymptomatic and 3.2% symptomatic branch occlusion rates at 6 months [56]. Interestingly, vision outcomes appear superior with FD use for paraclinoid aneurysm compared to clipping and coiling modalities [57], a finding reflected in the analysis of neuro-ophthalmologic outcomes at 6 months following PED placement, where 64% of patients with baseline deficits improved while only 5% of patients developed a new deficit [58]. Fusiform aneurysms of the basilar artery, especially those with partial thrombosis, are a special consideration for FD placement due to the devastating consequences of brainstem perforator infarctions. Thus, judicious use of flow diversion for these lesions is recommended [59]. 

FD placement across the ICA terminus produces jailing of the anterior cerebral artery (ACA) A1 segment. The fate of jailed A1 segments appears dependent on the presence or absence of adequate collateral flow through an anterior communicating artery (ACoA). In one study evaluating the effects of jailing A1 for distal ICA IAs, at a mean 14-month follow-up, 31% and 52% of jailed A1 segments with a patent ACoA were occluded or narrowed, all of which were asymptomatic [60].

Key principles pertaining to the fate of jailed side branches have emerged based on this and additional work. Perforators remain patent due to a sump effect [61]. Side branches either stay open if terminal with poor collaterals supplying their distal vascular territories (e.g., AChA, M1 lenticulostriates, A1 with insufficient ACoA collateral flow) or occlude without symptoms in cases of adequate extracranial–intracranial anastomoses or intracranial collaterals (e.g., OphA with robust external carotid artery collaterals, PCoA with dominant P1, A1 with sufficient ACoA collateral flow) [59]. While a detailed explanation of the technical nuances is beyond the scope of this review, appropriate sizing and minimizing the number of FD devices deployed across a segment should be pursued to reduce metal coverage across the jailed ostium [62,63,64]. Finally, initiating and adhering to a therapeutic antithrombotic regimen to prevent in-stent thrombosis is crucial. Clopidogrel non-responders should be identified and switched to another agent (e.g., Prasugrel or Ticagrelor), and patients should be educated on the importance of medication compliance [65]. 

## 5. Landmark Trials—PITA and PUFS

### 5.1. Pipeline Embolization Device for Intracranial Treatment of Aneurysms (PITA) Trial

The earliest large-scale accounts of PED use for the treatment of WNAs emerged in 2009 from groups out of Buenos Aires. Preliminary results from a series of 63 small, large, and giant unruptured intracranial aneurysms (UIAs) were promising, with complete angiographic occlusion occurring in 56%, 93%, and 95% of aneurysms at 3, 6, and 12 months, respectively [66]. Safety outcomes appeared reassuring, and no UIAs underwent recurrence. Fueled by these encouraging results and accompanying positive results from the International Subarachnoid Hemorrhage Trial (ISAT) and Barrow Ruptured Aneurysm Trial (BRAT) demonstrating advantages of endovascular therapies over surgical clipping in certain ruptured IA scenarios, the PITA trial was commenced in 2007. 

PITA was a prospective, single-arm trial of PED Classic that enrolled 31 patients in Europe and South America harboring 31 wide-neck and previously treated/failed aneurysms [67]. Ophthalmic ICA segment (61.3%) aneurysms were most commonly represented, with 71% of IAs meeting WNA criteria. At 6-month follow-up, 93.3% of IAs (28/30) were completely occluded, and the majority of treatments were without evidence of parent vessel stenosis. One patient displayed asymptomatic, mild (25–50%) parent vessel stenosis. The authors concluded that complex IA reconstruction with FDs was feasible and demonstrated angiographic and clinical outcomes superior to conventional coil-based endovascular techniques. 

### 5.2. Pipeline for Uncoilable or Failed Aneurysms (PUFS) Trial

The pivotal study leading to PED FDA approval came a year later, in 2008, with the enrollment of patients into the North American PUFS trial [7]. The PUFS trial was a prospective, single-arm trial of PED Classic for large (>10 mm) and giant (>25 mm) unruptured WNAs of the ICA from petrous to superior hypophyseal segments, all of which were required to meet WNA criteria of aneurysm neck diameter ≥ 4 mm. Previously treated aneurysms could be included as long as the previous treatment did not include an endoluminal device. Patients were prescribed a DAPT regimen of aspirin 325 mg daily and clopidogrel 75 mg daily preoperatively and then for between 3 and 6 months postoperatively. Comprehensive neurologic and neuro-ophthalmologic exams were performed at baseline and interval periods up to 6 months post-treatment. A follow-up diagnostic cerebral angiogram was performed at 6 months to assess for the degree of aneurysm occlusion and parent vessel stenosis. The primary effectiveness endpoint was the rate of complete aneurysm occlusion without major (>50%) parent artery stenosis or adjunct use of a secondary embolic agent (e.g., coils). Angiograms were evaluated by an independent core lab of three interventional neuroradiologists. The primary safety endpoint was the incidence of major ipsilateral ischemic or hemorrhagic stroke (increase in NIHSS of ≥4 points) or neurologic death within 6 months following PED placement. 

The results from PUFS represented a landmark moment for flow diversion as an effective and safe treatment for IAs. A total of 108 aneurysms were enrolled, with an average fundus and neck diameter of 18.2 mm and 8.8 mm, respectively. Giant aneurysms represented 20.4% of the cohort, and 7.4% (8/108) were deemed failed after undergoing previous treatment. The most common aneurysm locations were cavernous (40.7%), followed by paraophthalmic (32.4%) and supraclinoid (9.2%). PED placement was technically successful in 99.1% (107/108) of procedures. Of the 106 IAs evaluated at 6 months, 73.6% of treated aneurysms met the primary endpoint. Reasons for failing to reach the primary endpoint included residual aneurysm fundus/dome or neck filling (13.2%), parent artery stenosis > 50% (1.8%), and death (2.8%). One aneurysm required additional embolization with coils (0.9%). The primary safety endpoint was seen in six patients: three for ipsilateral ischemic stroke, two for ipsilateral hemorrhagic stroke, and one death possibly attributed to neurologic origin for a combined rate of 5.6%. The spontaneous post-treatment rupture rate was 1.9%. PUFS featured a Bayesian analysis of primary endpoints with a prespecified threshold that effectiveness exceeded (50%) and safety did not exceed (20%). The posterior probability for primary effectiveness exceeding 50% was 0.999999, and that for primary safety not exceeding 20% was 0.999979. 

Despite limitations and criticisms of the study, such as a high proportion of female participants (88.9%) and lack of a control arm, the authors concluded that the use of PED for selected difficult-to-treat WNAs was effective and safe. Long-term follow-up of the original PUFS cohort showed progressive rates of complete IA occlusion with time, from 73.6% at 6 months to 86.8%, 93.4%, and 95.2% at 1, 3, and 5 years, respectively. Six patients (5.7%) required retreatment for residual aneurysm, and no previously occluded aneurysm demonstrated recanalization at any point. No delayed device-related strokes or deaths were reported past 6 months. Functional independence, as assessed by the modified Rankin Scale (mRS), was seen in 96.3% of patients at 5 years (mRS ≤ 2) [42,68]. The results from PUFS led to FDA premarket approval of the PED in 2011 for large and giant WNAs of the petrous through superior hypophyseal ICA segments [6], the first FD approved for use in the United States. 

## 6. Flow Diverter Advances after PITA and PUFS—Next-Generation PED and Expanded PED Indications

Despite encouraging results from PITA and PUFS, accounts of more frequent and previously unreported neurologic adverse events raised concerns over the safety of FDs for both on-label and expanding off-label indications. The Retrospective Analysis of Delayed Aneurysm Ruptures after flow diversion (RADAR) study reported a 1% (14/1421) delayed post-treatment IA rupture rate, but with all ruptures occurring in large IAs > 10 mm (2% of all large aneurysms) [69]. A meta-analysis of 1654 IAs by Brinjikji et al. found morbidity and mortality rates of 5% and 4%, respectively. Rates of ischemic stroke (6% overall) and perforator infarction (3% overall) were significantly higher in larger anterior and all posterior circulation aneurysms [70]. 

### 6.1. International Retrospective Study of the Pipeline Embolization Device (IntrePED) and Aneurysm Study of Pipeline in an Observational Registry (ASPIRe)

The IntrePED study retrospectively analyzed 906 IAs with variable morphology, location, and rupture status treated with PED Classic [71]. The study found a neurologic morbidity and mortality rate of 8.4%, with rates of spontaneous intracranial hemorrhage (ICH) of 2.4%, ischemic stroke of 4.7%, and post-treatment rupture of 0.6%. Mortality dropped to 5.7% when excluding patients with ruptured, dissecting, or fusiform aneurysms. Adverse events were more common in posterior circulation and larger anterior circulation aneurysms compared to smaller, anterior circulation aneurysms (neurologic morbidity and mortality: 16.4% for posterior circulation IAs vs. 4.8% for anterior circulation IAs < 10 mm, *p* = 0.01; ischemic stroke: 7.3% vs. 2.7%, *p* = 0.16; neurologic mortality: 10.9% vs. 1.4%, *p* < 0.01). 

In a large post-market prospective study on PED Classic safety, ASPIRe collected data on 207 unruptured IAs treated with PED [72]. Safety findings corroborated those seen in PUFS and IntrePED, with rates of long-term neurologic morbidity and mortality of 6.8% (c.f. 8.4% IntrePED, 5.6% PUFS), post-treatment delayed aneurysm rupture of 1.6% (c.f. 0.6% IntrePED, 1.9% PUFS), and non-aneurysmal ICH of 3.7% (c.f. IntrePED 2.5%, PUFS 4.7%). Collectively, these post-market studies reaffirmed the safety of PED Classic for anterior circulation IAs seen in PUFS, further supported the off-label use of PED for small < 10 mm anterior circulation saccular IAs, but cautioned the off-label use in ruptured, non-saccular, and posterior circulation IAs. 

### 6.2. Pipeline Flex and Pipeline with Shield Technology

As post-market experiences of the PED Classic began to surface, several improvements were being made to the PED device based on reports of difficulty in device delivery. Pipeline Flex [73], approved by the FDA in 2015, featured the same device as PED Classic but aimed to improve ease of use through an upgraded delivery system that allowed partial deployment and repositioning by re-sheathing [31]. Brasiliense et al. reported on 233 unruptured IAs treated with PED Flex and found a 100% successful deployment rate with low rates of technical events related to device deployment (2.4% 30-day morbidity and mortality, 6.8% asymptomatic intraprocedural events). Further modifications to PED came in 2015 with the introduction of Shield technology [33], a surface-modified version of PED Classic featuring a covalently bound PC endoluminal layer, mimicking the surface of erythrocytes, for reduced device thrombogenicity. 

The PFLEX study [33] was a prospective, single-arm trial of PED Flex with Shield technology for 50 unruptured IAs. The majority of IAs were small (<10 mm) and located along the ICA (94%). The addition of Shield technology demonstrated excellent clinical results compared to those seen with the PED Classic device in PITA, PUFS, ASPIRe, and IntrePED. Major stroke or neurologic death at 30 days and 1 year occurred in 0% of cases. Angiographic complete occlusion was seen in 76.3% at 6 months and 81.8% at 1 year, with only one case of parent vessel stenosis > 50% seen at any time point. El Naamani et al. compared another cohort of 50 unruptured IAs treated with PED Shield to 150 unruptured IAs treated with PED Classic/Flex at a single institution and found similar, non-inferior outcomes (PED with vs. without Shield technology intra-procedure complication rate: 0% vs. 4%, *p* = 0.727; peri-procedural: 4% vs. 6%, *p* = 0.701; complete occlusion at 6 months: 80.5% vs. 79.5%, *p* = 0.119; in-stent stenosis at 6 months: 14.6% vs. 14.2%, *p* = 0.927; retreatment: 0% vs. 5.3%, *p* = 0.097) [74]. A 2024 meta-analysis of 1680 unruptured IAs treated with PED Shield found rates of 6–24 month complete occlusion of 79%, in-stent stenosis > 50% of 0.8%, major ischemic stroke of 1.2%, and intracranial hemorrhage of 1.5%, confirming the excellent outcome and safety performance of the PC surface-modified PED [75]. Based on further convincing results of the post-market, prospective, multi-center SHIELD study [76], Pipeline Flex with Shield Technology was granted FDA approval in 2021.

### 6.3. Antiplatelet Regimens and Hemorrhagic and Thromboembolic Complications in the PFLEX Study

While all patients in PFLEX were initiated on DAPT prior to FD placement and remained on DAPT at 30 days post-procedure, by 1 year, only 20% of patients remained on DAPT. Conversely, 12% of patients were able to stop AP therapy entirely at 12 months. The reduced duration and magnitude of AP regimens observed in PFLEX were profound, given known issues of major hemorrhagic complications with prolonged DAPT use and thromboembolic complications (TECs) with variability in clopidogrel responsiveness [77]. For instance, major hemorrhagic complications from DAPT regimens (i.e., intracerebral hemorrhage, internal visceral bleeding, blood loss requiring transfusions) have been reported in 3.7–5.1% of patients in large cardiac stent series [78]. Non-negligible rates of minor, superficial, “nuisance” bleeding (e.g., easy bruising, difficult to stop bleeding with minor cuts) with long-term DAPT use, 27% in the one FD series, is another known problem [79]. In addition to permitting shorter DAPT courses, the ability to initiate single antiplatelet therapy (SAPT) with surface-modified FDs appears feasible. One meta-analysis of 295 IAs treated with surface-modified FDs and SAPT showed a reduction in intracranial hemorrhagic complications to 0.1% with an overall TEC rate of 7.6%. Subgroup analysis based on the AP agent used showed lower TEC rates for prasugrel (2.4%) and clopidogrel (4.2%) compared to aspirin (20%), suggesting a role for ADP-receptor antagonist monotherapy [80]. 

### 6.4. Prospective Study on Embolization of Intracranial Aneurysms with the Pipeline Device (PREMIER) and Expanded FDA Approval of PED

With strong evidence backing safety and efficacy for small and medium WNAs and technological improvements in device delivery came expanding on-label indications for PED use. The PREMIER trial was a 2014–2015 multicenter, single-arm study of PED Classic and Flex for the treatment of unruptured small and medium-sized WNAs of the intracranial ICA up to the carotid terminus and intradural vertebral artery (VA) up to and including the posterior inferior cerebellar artery (PICA). Inclusion criteria required adequate platelet responsiveness to clopidogrel (PRU 60-200). A total of 141 IAs were treated in a cohort similar to PUFS, consisting of 87.9% women. The median aneurysm size was 4.6 mm (84.4% small < 7 mm, 15.6% medium 7–12 mm) with a median neck size of 3.7 mm and predominately saccular morphology (96.5%, c.f. 3.5% fusiform). The majority of aneurysms treated were of the ICA ophthalmic segment (74.6%), followed by the ICA communicating segment (14.2%) and VA (5%). Technical success was achieved in all but one case (99.3%). The primary endpoint of complete occlusion without parent vessel stenosis > 50% or retreatment was seen in 76.8% and 83.3% at 1 and 3 years, respectively. Combined major morbidity and mortality rates were 2.1% and 2.8% at 1 and 3 years without a single instance of delayed aneurysm rupture. Results from PREMIER led to FDA expanded on-label approval of PED in 2019 to include small and medium-sized WNAs of the ICA up the carotid terminus. 

## 7. Rise of Competitor Endoluminal Devices and Emergence of Intrasaccular Flow Diverters 

### 7.1. Stryker Streamline and the Surpass Intracranial Aneurysm Embolization System Pivotal Trial to Treat Large or Giant Wide Neck Aneurysms (SCENT) Trial

With the achievements of FD in the United States and abroad in the 2010s came additional competitor devices aiming to replicate and improve on the success of the Medtronic Pipeline and Balt Silk devices. Surpass Streamline (SS, Stryker Neurovascular) is a braided elgiloy stent with a variable number of 48–96 strands based on device diameter [37]. The stent is designed to maintain mesh density and diamond cell shape across a wide range of arterial diameters, providing consistent flow diversion across the aneurysm neck irrespective of parent vessel size. The delivery system comes preloaded with an inner “pusher” catheter and an outer “delivery” catheter. The addition of a central microwire for a tri-axial “over the wire” approach provides better system stability, but there is a noted risk of distal vessel perforation from the microwire. 

SS was evaluated for the treatment of large and giant wide-neck aneurysms of the intracranial ICA up to the carotid terminus in the SCENT trial. SCENT met the primary efficacy and safety endpoints at 12 months with a 62.8% complete occlusion rate without major (>50%) parent vessel stenosis or retreatment and 8.3% combined major ipsilateral stroke or neurologic death [8]. Results from SCENT led to FDA approval of SSS in 2018, only the second FD to obtain premarket approval in the United States. SCENT was notable for a higher proportion of saccular IAs in the communicating segment of the ICA (35.5%) and IAs with fusiform morphology (8.3%). Additionally, the number of devices deployed was considerably lower than in other FD pivotal studies, with 88.3% of patients requiring only a single device for successful IA treatment. A meta-analysis of 1218 PED-treated IAs from the PUFS, ASPIRe, and IntrePED trials showed singular FD use in only 62.9% of cases [81], while a direct propensity score-matched study of 44 PED- vs. 44 SS-treated IAs reflected similar findings with an average of 1.75 vs. 1 (*p* < 0.001) devices used, respectively [82]. As previously discussed, metal coverage across perforators is largely driven by the number of devices deployed across the vessel ostium. While how *effectively* multiple devices are employed is arguably the most important determinant of aneurysm vs. side branch metal coverage [83], the ability to adequately treat the target IA with fewer devices is theoretically preferable. 

The second-generation device Stryker Surpass Evolve (SE) is a low-profile version of SS featuring upgrades to both the stent and delivery systems. SE employs fewer total strands while maintaining a consistent mesh density with the earlier-generation SS and abandons the preloaded delivery system of SS, allowing for improved maneuverability and wall apposition at its distal ends. While the formal clinical trial of efficacy and safety for SE remains ongoing, off-label multicenter accounts report comparable occlusion and morbidity/mortality rates to other next-generation FDs [84,85].

### 7.2. MicroVention Flow-Redirection Endoluminal Device (FRED) and the US Pivotal Trial

The FRED (MicroVention) is a dual-layer, self-expanding, nitinol braided stent featuring an interconnected 48-strand inner layer and a 16-strand outer layer. The dual-layer architecture is designed to enhance the navigability and wall apposition of the device in tortuous anatomy with a high-porosity outer layer similar to coil support stents while maintaining optimal flow diversion effects with a high-metal-coverage inner layer. The outer layer also provides the “anchor” with no flow-diverting properties, which has to be taken into account. In comparison to other FDs, FRED’s advantage is its more straightforward delivery, allowing for precise deployment. Depending on size, FRED is delivered through a 0.027” or 0.021” microcatheter. FRED demonstrated safety and efficacy comparable to other FD devices in two large European studies and the US Pivotal trial of FRED with 1-year rates of complete aneurysm occlusion of 62.9–91.3%, perioperative permanent morbidity of 0.8–4.1%, and neurologic death of 0.7–1.9% [9,86,87]. Based on the US pivotal trial results, the FDA approved FRED for the treatment of saccular WNAs and fusiform IAs of the petrous ICA through the carotid terminus in 2019. 

A real-world North American multicenter experience of FRED for 133 IAs, however, expressed concerns over efficacy and safety not seen in the previous trials [88]. At a last median follow-up of 7.0 months, residual aneurysm filling was seen in 35.6% of cases with rates of moderate to severe parent vessel stenosis (>50%) of 8.1%, parent vessel occlusion of 9.1%, covered branch occlusion of 9.5%, and FRED-related and symptomatic complications of 22.4% and 12.9%, respectively. The authors postulate that the suboptimal efficacy and safety of FRED may be related to the dual-layer design, whereby (1) the outer high-porosity layer prevents optimal flow diversion at the aneurysm neck and a weaker drop in intra-aneurysmal pressures, (2) the cumulative effect of dual layers results in greater metal coverage across the device, leading to higher rates of in-stent stenosis and perforator occlusion, and (3) the creation of microthrombus between layers results in increased intimal hyperplasia and subsequent higher rates of in-stent stenosis and parent vessel occlusion. In a retrospective, propensity-matched analysis of FRED vs. PED, El Naamani et al. also showed lower unadjusted 6-month occlusion rates (51.5% vs. 74.7%, *p* = 0.017) and a near double rate of in-stent stenosis with FRED (15.2% vs. 6.9%, *p* = 0.172); however, the significance of the efficacy outcome was lost with propensity score matching [89]. Further large-scale, randomized, prospective comparisons between single- and dual-layer FD designs are needed. 

A surface-modified version, FRED with X-technology (poly 2-methoxyethyl acrylate coating), was FDA-approved in 2021. Compared to the classic FRED, preliminary real-world results of FRED X are promising, with improved efficacy and lower rates of moderate–severe and symptomatic in-stent stenosis (<6%) and thromboembolic complications [90]. 

### 7.3. Intrasaccular Flow Diversion with the MicroVention Woven EndoBridge Device (WEB) and the WEB Intrasaccular Therapy (WEB-IT) Trial

Wide-neck bifurcation aneurysms (WNBAs) can be difficult to treat due to balancing the consideration of targeting complete aneurysm occlusion while maintaining the patency of two or more branch vessels. WEB is an ISFD designed specifically for WNBAs [91]. Device architecture includes barrel-shaped and spherical designs comprising between 114 and 216 nitinol/platinum braided wires joined at each end by radiopaque platinum markers. The device is attached to a flexible delivery wire, delivered through a microcatheter, released with an electrothermal mechanism, and is fully re-sheathable once deployed. WEB ranges in size from 3 × 2 mm^2^ to 11 × 9 mm^2^; as such, their use is restricted to small and medium-sized IAs. When properly sized, the WEB device creates a fast seal at the aneurysm neck, leading to intra-aneurysmal stasis and thrombosis. Undersizing produces a dog-ear remnant along the neck, while oversizing predisposes the treated aneurysm to branch vessel impingement and protrusion of the device into the parent vessel; modest intentional oversizing of the device width by 1–2 mm, however, is recommended to allow for optimal aneurysm wall apposition where the risk of recurrence is highest. Metal coverage is considerably greater compared to endoluminal FDs, attaining near 100% metal coverage centrally near the radiopaque markers and decreasing radially to 60–65% at the device periphery. The increased mesh density enables enhanced intra-aneurysmal flow reduction and later neointimal formation across the device–neck interface. An advantage of WEB is that antithrombotic therapy is not required when appropriately sized with mesh placement confined to the aneurysm sac. Depending on the selected size, WEB may be delivered through 0.017-inch to 0.033-inch ID catheters. 

WEB was prospectively evaluated in three European studies prior to the commencement of a pivotal trial in North America [92,93,94]. Collective results from 169 WNBAs enrolled in these three European studies showed favorable efficacy and safety outcomes compared to other contemporary endovascular treatment options [95,96], with one-year complete and adequate occlusion rates of 52.9% and 79.1%, a low periprocedural complication rate of 1.2% with no mortality, and a retreatment rate of 6.9% [97]. 

WEB-IT was a prospective, multicenter, single-arm, North American premarket approval trial of WEB for WNBAs. WEB-IT enrolled 150 patients with both unruptured (94%) and low-grade ruptured (n = 9, 6%; Hunt and Hess I and II) saccular WNBAs of the anterior (60.7%) and posterior (39.3%) circulations at the basilar apex (39.3%), MCA bifurcation (30.0%), ACoA complex (26.7%), and ICA terminus (4.0%) locations. AP therapy was not mandated, but the most common regimen involved DAPT at the time of the procedure (69.3% vs. SAPT 27.3% vs. none 3.3%) followed by a transition to SAPT by 30 days post-procedure (56.0% vs. DAPT 31.3% vs. none 12.7%). Half of all WEB-treated patients had all AP therapy discontinued at one year. A unique angiographic occlusion system, the WEB Occlusion Scale (WEB-OS), was developed and validated for core laboratory adjudication of primary effectiveness endpoints in WEB trials [91]. The primary effectiveness endpoint was the proportion of patients with complete occlusion (WEB-OS A and B, i.e., complete WNBA fundus and neck occlusion) without need for retreatment, recurrent SAH, or significant (>50%) parent vessel stenosis at 1 year. 

The primary endpoint was met in 77/153 WNBAs (53.8%), with most WNBAs showing progressive occlusion and stability at 6 and 12 months, respectively. WNBA recurrence was seen in 5.0% (7/141) and 11.5% (15/131) at 6 and 12 months, respectively, and 9.8% (14/143) required retreatment. There were no incidences of significant parent vessel stenosis or post-treatment aneurysm rupture. Primary safety outcomes were seen in 0.7% perioperatively up to 30 days and in 0% from 30 days to 1 year [98]. There were no deaths. There were a few minor ischemic (4.7%) and hemorrhagic (1.4%) periprocedural complications, all of which returned to baseline except for one patient. Two intraprocedural SAHs occurred with resultant minimal symptoms; there were no delayed ruptures after 30 days post-procedure. WEB for WNBAs showed durability up to 5 years in WEB-IT with long-term complete and adequate occlusion rates of 58.1% and 87.2% and an overall retreatment rate of 15.5% [99]. A post-market North American multi-center experience of WEB for 76 WNBAs found complete and adequate occlusion rates of 49% and 78% [100], and a systematic review of over 400 WEB-treated WNBAs found larger aneurysm size and neck width to be significant predictors for incomplete aneurysm occlusion [101]. WEB for the treatment of WNBAs was granted FDA premarket approval in 2019. 

While SS, FRED, and WEB represent major competitor takes on the classic PED device, each with unique features related to intrinsic design and deployment systems, the rise of flow diversion has led to many other devices in the North American and Global markets. While an extensive description of all available devices is beyond the scope of this review, a number of dedicated accounts on this topic are available [102]. Other FD devices currently in use but without formal FDA approval include Phenox p64 and p48 MW [103], Balt Silk and Silk Vista [104], MicroPort Tubridge [105], and Acandis Derivo [106,107]. Many others are in development in the proverbial pipeline.

## 8. Future Directions—Expanding Indications beyond the Circle of Willis

While current approved indications for FD therapy include WNAs up to the carotid terminus, the use of FDs for IAs distal to the ICA has gained popularity. A major historical limitation to using classic FD devices like PED for IAs beyond the Circle of Willis has been the need for larger inner diameter (ID) microcatheters (0.027-inch) for device deployment. These devices and their associated delivery systems, designed for proximal parent vessels sized 4.0–5.0 mm in diameter, are more challenging to navigate through distal intracranial vascular anatomy due to greater tortuosity and smaller caliber (usually <2.5 mm diameter) of parent vessels harboring IAs. Two of the earliest reports of FDs for distal IAs involved the use of PED Classic within the M1, M2, A2, and P2 segments [108,109]. In 93 treated IAs, at a median follow-up of 6–10.7 months, complete aneurysm occlusion was seen in 75–83% with good clinical outcomes (mRS 0-2) in 95.0–95.4%. These early studies concluded that FD therapy for distal IAs was feasible with acceptable occlusion and good clinical outcome rates for IAs not amenable to traditional surgical or endovascular techniques. 

### Emergence of Low-Profile Flow Diverters for Distal Aneurysms

A number of low-profile systems were developed and brought to the market in the late 2010s to improve the deliverability of FD devices to distal IAs. These systems allow for the use of smaller ID microcatheters (0.017–0.021 inches) to deliver micro-FD devices into parent vessels of sizes 1.5 to 3.0 mm. 

FRED Jr (MicroVention) is a low-profile version of FRED, featuring a double-layer, braided nitinol stent with deliverability through a 0.021-inch ID microcatheter for IAs with parent vessel diameters from 2.0 to 3.0 mm. Like FRED, the inner layer of FRED Jr has lower porosity with 36 wires to enhance flow diversion effect. The outer layer has greater porosity with 16 wires, similar in design to the coil support device LVIS by MicroVention, to enhance deliverability and wall apposition. Möhlenbruch et al. reported pivotal results from a multicenter, observational study of FRED Jr for forty-seven mostly unruptured IAs (one subacute SAH) with varying morphology (saccular: thirty-five; fusiform/dissecting: nine; giant: two; blister: one) and previous treatment status (five coils alone, four clipping, two flow diversion with PED and Silk) [110]. Parent vessel diameter ranged from 1.4 to 3.6 mm (median 2.4 mm), and all saccular IAs met WNA criteria. Complete and near-complete occlusion was progressive with rates of 66%, 78%, and 100% at 1, 6, and 12 months, respectively. There were no retreatments. Device deployment was achieved in all cases. Periprocedural complications occurred in 7.1% (3/42 patients), including one disabling stroke, one minor stroke with recovery by discharge, and one TIA. A recent meta-analysis of seven studies including 227 patients with 244 IAs treated with FRED Jr found pooled complete and near-complete occlusion estimates of 55.9% and 62.5% at 6 months and 63.8% and 74.2% at 12 months, respectively [111]. Retreatment was seen in 0.5% (1/197). Periprocedural stroke morbidity was 5.7% (13/227), with no periprocedural mortality. At final follow-up, good clinical outcomes (mRS 0-2) were seen in 97.6% (164/168). Overall, the FRED Jr micro-FD has shown favorable periprocedural efficacy and safety outcomes with durable medium-term follow-up compared to previous studies of higher-profile FD systems for distal IAs with parent vessel diameter < 3 mm.

Silk Vista Baby (Balt) is another low-profile FD designed for parent vessels of 1.5–3.5 mm in diameter and the only such device compatible with a 0.017-inch ID microcatheter. SVB is composed of braided DFT wires, consisting of a radiopaque platinum core surrounded by an outer nitinol shell, for enhanced visualization while maintaining expandability at the target location [112]. There are reports of successful SVB off-label use with even smaller 0.0165-inch microcatheters (e.g., Stryker Excelsior SL-10), which may be better suited for IAs of the M2-3 and A2-pericallosal segments [113]. A systematic review of 173 IAs of the ACA, MCA, and ACoA treated with SVB found pooled estimates of complete and near-complete occlusion in 72.7% at short-term follow-up (range: 0–10.6 months) [114]. Successful treatment was seen in parent vessel diameters as small as 0.9 mm. Major stroke (1.2%) and neurologic death (1.8%) were low despite a high proportion of acutely or previously ruptured IAs (29%). The favorable “lowest-profile” nature of SVB has led to its use in other traditionally difficult-to-treat endovascular locations, with Benalia et al. reporting a series of 15 true PICA aneurysms (i.e., distal to the ostium), all of which achieved 100% complete occlusion at 6 month follow-up with only one treatment-related adverse event [115]. 

As devices, surface modifications, and delivery systems continue to evolve, the expanded use of FDs for distal IAs—including those of the posterior circulation [116] and with parent vessels < 2.0 mm diameter [117]—will undoubtedly continue to proliferate. 

## 9. Future Directions—Expanding Indications for Ruptured Aneurysms 

### 9.1. Challenges of Endoluminal Flow Diverters for Ruptured Aneurysms

The implementation of FD therapies for ruptured IAs poses challenges due to two main factors. First, all FDs placed within parent vessels require administering antithrombotic medications to prevent in-stent and distal thromboembolic complications. Even with the development of endoluminal surface modifications such as Shield (phosphorylcholine, Medtronic), X (poly 2-methoxyethyl acrylate, MicroVention), and HPC (glycan-based multilayer hydrophilic polymeric coating, Phenox; Bochum, Germany) which provide covalently bound biomimetic substrates to the bare metal surface of devices, to date, all FDs still mandate DAPT [118]. As such, placement of FDs in the acute setting of aneurysmal SAH imposes additional risks in terms of new and worsening bleeding from aneurysm re-rupture or during procedures such as external ventricular drain placement. The need for AP reversal with life-threatening intracranial hematoma expansion further increases the risk of device-related thromboembolic complications. 

Second, while varying degrees of intrasaccular stasis are often seen after the deployment of FDs, with rates of immediate complete angiographic occlusion of 14.5–32% in ruptured cases [119,120], there remains a period of time where the aneurysm remains at risk of re-rupture from incomplete dome protection and neointimal formation. An early meta-analysis by Madaelil et al. of 126 ruptured IAs found rates of aneurysmal rebleeding after FD placement of 5% (6/126); sub-analysis showed re-rupture of large IAs > 2 cm as the principal driver of increased rebleeding following FD therapy (re-rupture rates of 57% (4/7) for IAs > 2 cm compared with 2% (2/94) for IAs < 2 cm (*p* < 0.001)) [121]. Adjunctive coiling was not found to reduce rebleeding (*p* = 0.672). In a later 2018 meta-analysis of 223 patients undergoing FD treatment for IAs, the re-rupture rate was 4%. Both meta-analyses found that re-rupture rates were highest during the first 24–72 h after retreatment, and no rebleeding occurred after the first week following treatment. Subsequent reports have found conflicting rates of aneurysm re-rupture, with one series of 76 ruptured IAs treated with standalone flow diversion showing no episodes of re-hemorrhage, while an updated 2022 meta-analysis of 318 FD-treated ruptured IAs found pooled rates of rebleeding as high as 12% (95% CI, 8–15%) [120,122]. 

The 4–5% rebleeding rate in the two early meta-analyses is higher than the endovascular rebleeding rate seen in ISAT (2.7%), where standalone coiling was employed [123]. Furthermore, while complete and near-complete occlusion rates in the Cagnazzo meta-analysis were 88.9% at a mean follow-up of 9.6 months, there was a significant rate of treatment-related complications (17.6%), a 2-3-fold increase seen from the unruptured PED studies (5.6–8.4% in PUFS/IntrePED/ASPIRe). As such, endoluminal FD therapy for ruptured IAs has largely been limited to saccular lesions where traditional surgical clipping and endovascular coiling are unlikely to be successful or for many complex dissecting, fusiform, or blister aneurysms where no better treatment option exists [124]. The latest 2023 AHA/ASA Guidelines for Aneurysmal SAH reflect these findings, providing the following recommendations [125]:

- FD use is reasonable in aneurysmal SAH cases non-amenable to surgical clipping or endovascular standalone coiling (class 2a/moderate strength recommendation, benefit outweighs risk, class C limited data level of evidence based on observational/registry data and meta-analysis).
- FDs should not be used in cases where surgical clipping or endovascular standalone coiling are likely to provide effective treatment due to excessive morbidity and mortality associated with FD use (class 3/harm recommendation, class B non-randomized level of evidence).
- There are insufficient data on reduced thrombogenicity endoluminal devices for recommendation.

Given the risks of endoluminal FD placement in the acute phase of aneurysmal SAH, three main FD-based treatment strategies have prevailed. First, the “plug and pipe” staged treatment approach described by Howard et al. can be employed, where acute-phase coil embolization of ruptured IAs for dome protection is followed by delayed FD placement after acute SAH convalescence [44]. Second, if acute-phase endoluminal FD placement is anticipated, then adjunct surgical therapies such as external ventricular drain (EVD) or lumbar drain for treatment of manifest or anticipated hydrocephalus should be performed prior to AP administration [124,126]. In a meta-analysis of ventriculostomy-related hemorrhages in patients on AP therapy for endovascular treatment of acutely ruptured intracranial aneurysms, Cagnazzo et al. found that rates of ventriculostomy-related hemorrhages were significantly lower in those receiving intraprocedural AP when surgical drains were placed before initiation of AP compared to after (9.6% vs. 25.1%, *p* < 0.02) [127]. While there was an increased risk of EVD-related hemorrhages with any AP use (20.9% vs. 9%, *p* < 0.0001), most hemorrhages were small and asymptomatic, suggesting that the benefits of CSF diversion for aneurysmal SAH-associated hydrocephalus outweigh the increased procedural bleeding risks. Third, the use of intrasaccular FDs can be considered. 

### 9.2. Potential of Intrasaccular Flow Diverters for Ruptured Aneurysms

WEB provides advantages over endoluminal FD devices in the acute SAH setting by eliminating the absolute need for AP therapy. Still, intrasaccular manipulation is required for device placement, which may precipitate intraprocedural rupture. Al Saiegh et al. presented a post-market approval experience of WEB for 9 patients with acute aneurysmal SAH. They demonstrated feasibility in treating ruptured IAs of the anterior and posterior circulations across the spectrum of clinical severity [128]. There was one episode of intra-operative rupture, and one IA required additional stenting due to device prolapse into the parent vessel. The stented patient was the only patient to require AP therapy.

The CLinical Assessment of WEB device in Ruptured aneurYSms (CLARYS) study sought to determine the early rebleeding rate (first 30 days) of ruptured bifurcation aneurysms following WEB placement [129]. In 60 patients, WEB demonstrated excellent dome protection with no episodes of post-procedural rebleeding at 1-month and 1-year time points. Less than half of all WEB recipients required any intraprocedural AP therapy (43%), and only 30% remained on any AP at 1 month. The use of WEB in the acute SAH phase appeared safe with a procedure/device-related intraprocedural complication rate, including rupture, of 3.3%; WEB 1-month and 1-year morbidity and mortality were each 0%. Angiographic outcomes assessed at 1 year showed an adequate occlusion rate of 87%, with six aneurysms requiring retreatment [130]. 

In one of the largest meta-analyses to date, Xie et al. reported outcomes for 283 ruptured aneurysms treated with the WEB device [131]. Technical success was achieved in 99% of cases with an intraoperative rupture rate of 3%. Immediate complete and adequate occlusion was 38% and 98%, respectively. Perioperative rebleeding was 1%, less than the 4–5% seen with endoluminal FD and 2–3% seen with standalone coil embolization. Perioperative mortality was 9%; however, the study did not provide subgroup analysis in terms of SAH severity or complications. WEB treatment-related complications were seen in 7%. At latest follow-up, ranging from 6 to 13 months, complete and adequate occlusion was 61% and 91%, respectively, with retreatment performed in 6% of cases. 

While currently not a first-line treatment option for ruptured aneurysms, these early studies suggest that WEB has an acceptable safety profile and short- to medium-term outcomes in the acute treatment of ruptured IAs compared to other conventional endovascular therapies. 

## 10. Future Directions—Therapeutic Dilemmas, Emerging Technologies, and Cutting-Edge Research 

As more and more patients receive effective, safe, and durable treatment for a broader range of IAs with FD devices, with approximately 30% of all IAs now being treated with flow diversion, the demand for refined and improved FD products continues to rise. Pipeline Vantage, the latest-generation PED device, capitalizes on increased understanding of FD device design, utilizing a higher pore density (known as pics-per-inch) with thinner DFT wires to provide greater wall apposition during delivery and more robust scaffolding for neointimal growth [34,35,132]. New ISFDs such as Artisse (Medtronic) and Contour (Stryker) look to expand the range of endovascular treatment options for WNBAs and are currently being trialed in North American markets [133,134,135,136]. A new class of endovascular devices known as neck-bridging devices is also emerging onto the market. These devices minimize or eliminate intrasaccular mural contact and provide coverage only along the aneurysm ostium. The endovascular clip system (eCLIPS) (EVasc Medical Systems Corp) is a neck-bridging device that shares features of coil support stents and flow diverters while also permitting microcatheter passage through the device for added coiling [137]. 

### 10.1. Clinical Equipoise in the Treatment of Wide-Neck Bifurcation Aneurysms

While an extensive discussion of the subject is beyond the scope of this review, WNBAs represent treatment dilemmas for the neurointerventionalist. Clinical equipoise amongst the various endovascular modalities exists, with endoluminal FD, ISFD, balloon-assisted coiling, stent-assisted coiling [96], non-flow-diverting neck-bridging devices (Pulse Rider, pCONUS) [138,139], and flow-diverting neck-bridging devices (eCLIPS, pCANVAS) all representing viable treatment options with their associated advantages and disadvantages. Importantly, the superiority of endovascular treatment for IAs, as seen in studies such as BRAT and ISAT, has further been called into question, with a recent sub-analysis by Mascitelli et al. of WNAs in BRAT showing improved obliteration and retreatment rates with surgical clipping with non-inferior clinical outcomes compared to coiling [10]. A prospective, multicenter registry of microsurgical clipping vs. endovascular treatment for ruptured WNAs has shown similar outcomes, again questioning the superiority of endovascular modalities for this subset of IAs [140]. An important caveat in these traditional clipping vs. coiling trials is the underrepresentation or absence of FDs and ISFDs in the endovascular treatment arms. As a result, pragmatized studies assessing the utility of various surgical and modern endovascular treatment options for WNAs and WNBAs are ongoing. The results of these ambitious clinical trials, such as the Comprehensive Aneurysm Management (CAM) study and the Middle Cerebral Artery Aneurysm Trial (MCAAT), will hopefully provide greater clarity and guidance in the decision-making process for treating these challenging vascular lesions [141,142]. 

### 10.2. Beyond Surface Modifications—Development of Bioresorbable Flow Diverters 

Recent advances in surface modification technology have led to FDs with reduced thrombogenicity compared to traditional bare metal flow diverters. Given its highly effective antiplatelet effect in vitro, Phenox’s HPC surface modification is currently being used in a randomized trial of DAPT vs. prasugrel or ticagrelor-based SAPT as concomitant therapy for IAs treated with the p64 MW device [143]. Drug-eluting FDs represent another promising avenue for reducing thrombogenicity, with reports of a heparin-coated device yielding lower rates of thromboembolic events than uncoated devices in an animal model [144]. While research into more bio- and hemocompatible surface modifications for bare metal devices is ongoing, the emergence of bioresorbable flow diverters is anticipated to be the next major breakthrough in FD technology.

Currently, all FDs on the market are made of metal alloys such as nitinol or elgiloy, implants that remain embedded in the patient for the remainder of their lives. Bioresorbable stents, implants that fully degrade and disappear over time, have been explored extensively in the cardiac literature; however, only recently has the same research been applied to the cerebrovascular realm. Besides the finite lifespan and potential for reduced AP therapy durations, advantages of BRFDs over traditional metal alloy FDs include reduced thrombogenicity and vessel inflammation, lower risks of acute and chronic in-stent stenosis and perforator occlusion, attenuated metal artifact on follow-up CT/MR imaging, restoration of intrinsic vasomotor function, and compatibility with growing intracranial vessels in very young pediatric patients [52]. Leading candidates for BRFDs have included polymer-based materials such as polyglycolic acid and poly-L-lactic acid, as well as bioresorbable metals such as magnesium [145]. A number of critical questions remain regarding fundamental design principles for BRFDs, such as the requisite device lifespan to achieve adequate aneurysm exclusion, but the promise of BRFDs cannot be overlooked. 

### 10.3. Transcriptomics and Radiogenomics

Hereditable factors leading to the development of IAs are incompletely understood; however, the use of genome-wide association studies has furthered our understanding of the biological mechanisms of IA formation [146]. A limitation to understanding *local* gene and protein expression of the IA microenvironment has been the inability to obtain in vivo cerebrovascular specimens. Srinivasan et al. provide an excellent review of the current state of transcriptomics, i.e., the study of protein expression, as a tool for understanding the FD microenvironment [147]. Through the addition of liquid and endovascular biopsies, our ability to understand transcriptomic changes within aneurysms and parent vessels and the subsequent protein expression changes that ensue following FD implantation may improve our understanding of the biological underpinnings of aneurysm pathogenesis and FD treatment response. Similarly, radiogenomics, i.e., the linking of imaging phenotypes to genomic and transcriptomic profiles, will further advance translational and clinical studies, leading to more effective FD therapies and providing better, personalized treatment options for patients [148]. 

### 10.4. Robotics, Artificial Intelligence, and Machine Learning

Robotic-assisted neurovascular intervention is a nascent but rapidly expanding field. The CorPath GRX system (Siemens) is the first robotic system designed for neurointerventional procedures, allowing for the translation of manual movements from proceduralists into precise micromovements of intravascular guidewires and catheters. The remote nature of the robotic system means less radiation exposure for interventionalists and global access to high-level IA care [149]. Cancelliere et al. reported on six patients who underwent robotic-assisted neck-bridging device and FD treatment for IAs and found a 100% technical success rate without the need for bailout manual intervention. No patients experienced morbidity or mortality associated with the robotic procedure, and all demonstrated adequate occlusion at 1-year follow-up [149]. 

Machine learning and artificial intelligence remain hot topics in the endovascular domain. A recent study compared a predictive ML tool and a traditional logistic regression (LR) model for accuracy in predicting 6-month occlusion outcomes following FD treatment. For 667 IAs treated with PED, the ML tool outperformed the LR model (89% (AUC 0.94) vs. 62% (AUC 0.66), respectively) for correctly predicting aneurysm obliteration fate at 6 months [150]. At a recent roundtable discussion hosted by the American Stroke Association, a panel of hemorrhagic stroke clinical experts and industry leaders strongly recommended creating shared large-scale databases and implementing ML models to better predict IA rupture risk and guide treatment decisions [151]. 

## 11. Conclusions

Just as the introduction of Guglielmi detachable coils in the 1990s forever changed the face of cerebrovascular intervention, the emergence of FD devices over the last two decades has similarly revolutionized the endovascular management of IAs. In a paradigm shift from intrasaccular embolization to parent vessel remodeling as the primary mechanism for aneurysm exclusion, FDs have made many previously failed and difficult-to-treat IAs now curable. Currently, flow diversion holds FDA on-label approval for saccular and fusiform WNAs of all sizes of the ICA up the carotid terminus for endoluminal devices (Medtronic PED, Stryker Surpass, MicroVention FRED), and for saccular WNBAs of dome diameter 3–10 mm of the basilar apex, MCA bifurcation, ACoA complex, and ICA terminus locations for intrasaccular devices (MicroVention WEB) [152,153,154,155]. The AHA/ASA recommends against FD use in aneurysmal subarachnoid hemorrhage cases where surgical clipping or endovascular standalone coiling are likely to provide effective treatment due to excessive morbidity and mortality associated with their use [125]. 

PED and the landmark PUFS trial sparked the rise of flow diversion therapies in North America and abroad, leading to more sophisticated surface-modified devices as well as a whole new class of ISFDs. Despite the significant advances made for the endovascular treatment of unruptured WNAs, many areas for technological and decision-making improvement exist. Clinical equipoise for WNBAs and ruptured aneurysms remains steadfast [156]. The future possibilities of BRFDs, transcriptomics, ML models, and robotics systems for improved care for aneurysm patients are promising. The past, present, and future of flow diversion for intracranial aneurysms is a remarkable story of technological innovation leading to improved patient care, one that is worth continued watching. 

## Data Availability

No new data were created or analyzed in this study. Data sharing is not applicable to this article.
